# Associations between fishes (Actinopterygii: Teleostei) and anthozoans (Anthozoa: Hexacorallia) in epipelagic waters based on in situ records

**DOI:** 10.1111/jfb.70214

**Published:** 2025-09-05

**Authors:** Gabriel V. F. Afonso, G. David Johnson, R. Collins, Murilo N. L. Pastana

**Affiliations:** ^1^ Section for Natural Resources Virginia Institute of Marine Science, William & Mary Gloucester Point Virginia USA; ^2^ Department of Vertebrate Zoology – Division of Fishes Washington District of Columbia USA; ^3^ Florida Museum of Natural History University of Florida Gainesville Florida USA; ^4^ Museu de Zoologia da Universidade de São Paulo São Paulo Brazil

**Keywords:** blackwater photography, citizen science, commensalism, driftfish, filefish, jack, mutualism, pelagic invertebrates, pomfret, symbiosis, Western Atlantic

## Abstract

We formally describe the association of fishes and anthozoans in epipelagic waters, extending this relationship to beyond the benthos. In situ observations and photographs of *Aluterus schoepfii*, *Ariomma regulus*, *Caranx* cf. *latus* and *Brama* spp. swimming alongside or holding larval tube anemones (Cerianthidae and Arachnactidae) and larval zoanthids (Sphenopidae) were made during blackwater SCUBA dives off Palm Beach, Florida, USA, and off Punaauia, Tahiti, French Polynesia. We report and illustrate the behaviour of these interactions, and suggest an advantage for the anthozoans.

One of the most well‐documented symbiotic associations of the aquatic realm is the anemonefish‐anemone mutualistic interaction (Da Silva & Nedosyko, 2016). This association is formed by anemonefishes of the genus *Amphiprion* (Pomacentrinae; Fricke et al., 2025) and about 10 reported species of sea anemones (Anthozoa, Actiniaria – Dunn, 1981; Fautin, 1991). Those relationships are characterized by mutual benefits, including protection from predators, nutrient exchange and enhanced reproductive and lifetime fitness (Da Silva & Nedosyko, 2016). In the fish‐anemone association, species of anemonefishes are obligatory associated with sea anemones, but some species from 16 other fish families (Apogonidae, Blenniidae, Chaenopsidae, Chaetodontidae, Cirrhitidae, Psychrolutidae, Jordaniidae, Gobiidae, Haemulidae, Hexagrammidae, Holocentridae, Labridae, Labrisomidae, Pomacentridae, Scorpaenidae and Serranidae) associate facultatively with sea anemones (Allen et al., 2010; Elliott, 1992; Feeney et al., 2019; Hanlon & Kaufman, 1976; Randall & Fautin, 2002). In these facultative interactions, some fishes establish this association only during their juvenile phase, whereas others extend the association to adulthood (Feeney et al., 2019).

Associations between fishes and invertebrates are not limited to the benthos, but also occur in the pelagic environment (e.g., Griffin et al., 2019; Purcell & Arai, 2001). Fish‐jellyfish association, for example, has been extensively documented worldwide (e.g., Artüz & Tunçer, 2017; Bonaldo et al., 2004; Kondo et al., 2014; López Martínez & Rodriguez Romero, 2008; Rajkumar et al., 2014; Tilves et al., 2018; Villegas‐Ríos, 2009; Williams et al., 2001) and is regarded as a facultative symbiosis that occurs during the early life stages of fishes. Although jellyfishes are by far the most common hosts of juvenile pelagic fishes, associations also include siphonophores, comb‐jellies, nudibranchs and tunicates (Pastana et al., 2022, 2023; Purcell & Arai, 2001), which altogether comprise a non‐monophyletic group of pelagic invertebrates. Currently, 21 fish families are known to associate with pelagic invertebrates (Pastana et al., 2023).

In the context of recent discoveries of the early life history of marine organisms facilitated by blackwater diving (Nonaka et al., 2021), here we formally extend the fish‐pelagic invertebrate association to larval and juvenile fishes and anthozoans in planktonic stage. We report on four fish families (Monacanthidae, Ariommatidae, Bramidae and Carangidae) in association with three families of Anthozoa in the pelagic life stage: Arachnactidae and Cerianthidae (Ceriantharia), both popularly known as tube or tube‐dwelling anemones, and Sphenopidae (Zoanthidea), often referred to as encrusting anemones or zoanthids (Daly et al., 2003). The association reported here not only expands the number and diversity of pelagic invertebrates with which fishes associate but also expands the well‐known relationship between different types of anemones and fishes to the pelagic realm.

Observations described herein were made during night epipelagic (blackwater) drift dives. Photographs of one of the authors (R. Collins; Figure 1a,f) and Linda Ianniello (Figure 1b–d) were made using Nikon D series cameras equipped with a Nikon AF Micro‐NIKKOR 60 mm f/2.8D lens, inside Nauticam camera housings, along with various commercially available lights and strobes. Photographs of Figure 1a–d,f were captured off Palm Beach, Florida, USA, on drift dives ending at approximately 26° 46′ 12.0″ N, 79° 55′ 48.0″ W. The dives occurred between October 2018 and August 2023, between 7:30 and 10 PM local time (GMT −4), and at depths ranging from 8 to 15 m. Depth of the diving site varied from 167 to 228 m, and water temperature ranged from 24.4 to 30.5°C. Photograph in Figure 1e was made by Fabien Michenet, in Tahiti, French Polynesia, 2 km off the reef of Punaauia on 4 December 2014, at a depth of 20 m. Additional photographs of the same specimens or of other specimens that were not identified at the species level, or that represent species already reported here, were not reproduced in this paper.

**FIGURE 1:**
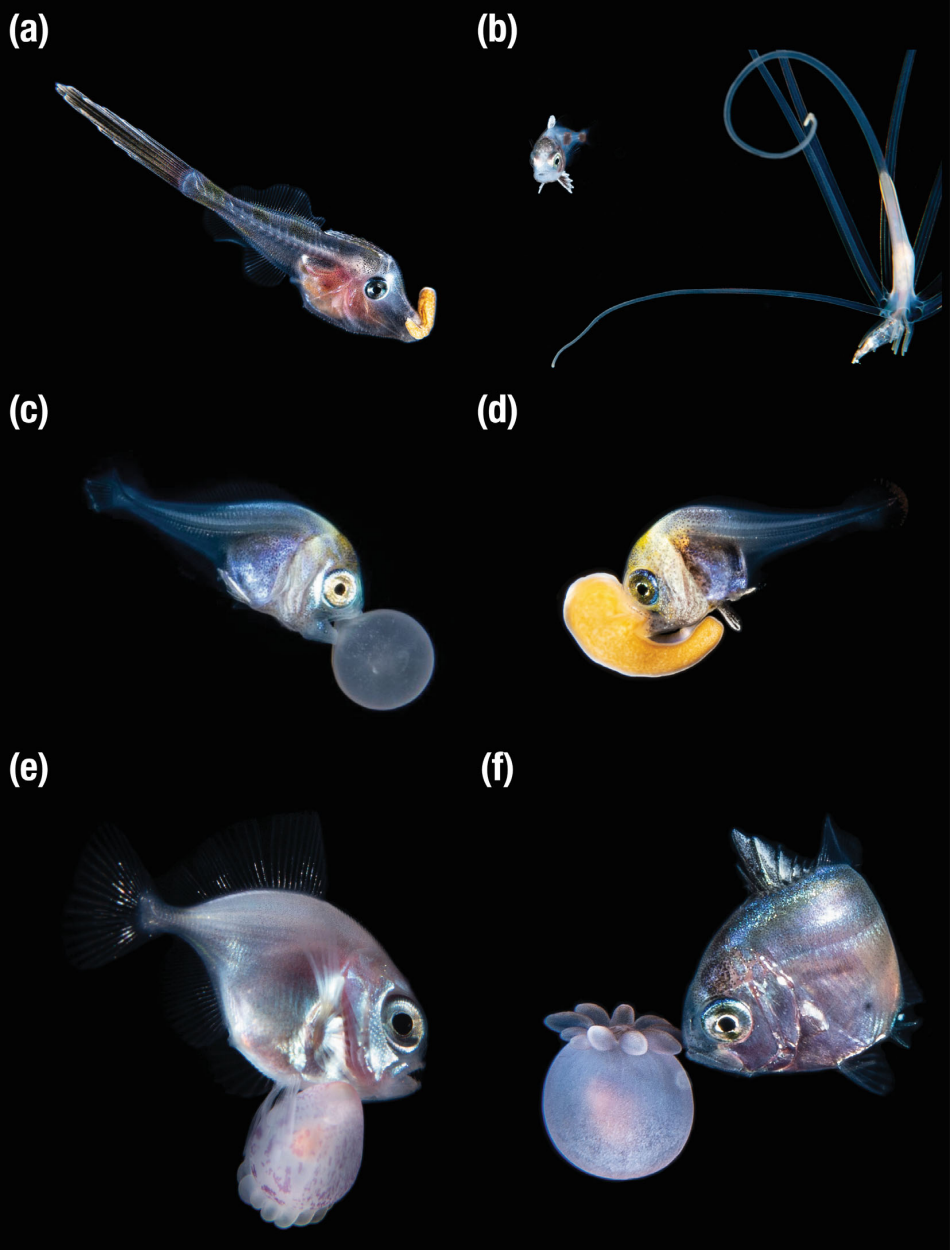
Fish‐anthozoan associations in epipelagic waters. (a) Juvenile *Aluterus schopfii* holding a *Palythoa* larva (Semper's larva), 18 June 2020; (b) Juvenile *Ariomma regulus* in association with a larval tube anemone (Ceriantharia), 5 January 2023, mirrored vertically; (c) Larval *Brama brama* holding a larval tube anemone *Isarachnanthus maderensis*, 6 April 2019, mirrored vertically; (d) Larval *B. brama* holding a *Palythoa* larva (Semper's larva), 20 October 2018; (e) Juvenile *Brama myersi* ‘riding’ an unidentified larval anthozoan, 4 December 2014; (f) Juvenile *Caranx* cf. *latus* in association with a larval tube anemone (Cerianthidae), 31 August 2023.

Fish capture and experimentation were not performed, and the research did not inflict harm to the fishes. The *Palythoa* specimen (Figure 1a) was collected and deposited in the Florida Museum of Natural History Invertebrate Zoology (FLMNH IZ) collection as UFID 14083. The Cerianthidae specimen (Figure 1f) was collected and deposited in the FLMNH IZ collection as UFID 16505. No permits were required to conduct this study.

Taxonomic identification of specimens was based on locality information of photographs, comparisons with literature descriptions (e.g., Berry, 1959; Berry & Vogele, 1961; Lamkin, 2005; Laroche et al., 1984; Laroche et al., 2005; Last & Moteki, 2001; McKenney, 1961; Moser & Mundy, 1996; Richards, 2005; Zapfe & Lyczkowski‐Shultz, 2005) and with museum specimens. Counts were made by hand with the aid of Adobe Photoshop's counting tool, and sizes of anemones were estimated using the ruler tool. Meristic and morphometric data obtained from the photographs include the following: pattern of body pigmentation, head and body shapes, number and pattern of spines on the preopercle and number of dorsal, anal and pectoral‐fin rays.

Based on our observations, fishes of four families associate facultatively with anthozoans in pelagic waters: Ariommatidae, Bramidae, Carangidae and Monacanthidae (Figure 1).

The juvenile monacanthid (Figure 1a) is estimated to be 19.5 mm standard length (SL), and can be identified as *Aluterus schoepfii* based on the presence of a long, barbed dorsal‐fin spine, minute spine‐covered scales and elongate body form. Other characters that further corroborate this identification include the presence of 36 dorsal‐ and 39 anal‐fin rays, flatly curved ventral profile and relatively shallow body depth (Berry & Vogele, 1961; Zapfe & Lyczkowski‐Shultz, 2005). The specimen was observed holding in its mouth a larval zoanthid of the family Sphenopidae, also known as Semper's larvae, possibly a species of *Palythoa* (James Reimer, personal communication; Martin & Koss, 2002), 5 mm in total length (TL). Adult specimens of some species of *Palythoa* produce a powerful toxin (Ashwood et al., 2020). However, it is not known whether the larvae produce palytoxin. If the larval *Palythoa* produce palytoxin, this would support the idea that early‐stage fishes associate with anthozoans in epipelagic waters for protection (see below).

The juvenile ariommatid (Figure 1b) can be identified as *Ariomma regulus* based on a notched dorsal fin, 15 branched dorsal‐fin rays and body colouration consisting of vertical bars (Lamkin, 2005; McKenney, 1961; Pastana et al., 2023). The colour pattern allows us to estimate the specimen to be around 10 mm TL (McKenney, 1961). The specimen was observed swimming in close proximity to a larval tube anemone, tentatively identified as *Isarachnactis* sp. (Cerianthidae).

Larval bramids (Figure 1c,d) are identified as *Brama brama* based on a large, round and heavily pigmented head and gut, and a long, slender tail with little pigmentation, as well as yellow hues on head, dorsal, anal and caudal fins (Richards, 2005). Based on notochord development and literature description (Richards, 2005), specimen of Figure 1c is at flexion stage and estimated to be 5–6.5 mm SL. This specimen was observed holding a tube anemone, tentatively identified as *Isarachnanthus maderensis* (Arachnactidae). Six similar specimens in the collection of the FLMNH range in size from 3 to 5 mm body length (BL). Figure 1d displays a *B. brama* in its preflexion form, estimated to be less than 5 mm SL (Richards, 2005), and holding a zoanthid larva (estimated ~3.5 mm TL).

The juvenile bramid in Figure 1e is identified as *Brama myersi* based on the compressed body, blunt, rounded head, oblique mouth and presence of 37 dorsal‐fin rays (Moser & Mundy, 1996; Richards, 2005), as well as an acute ventral profile at the anal‐fin base (Last & Moteki, 2001). Estimate of the total length of the photographed *B*. *myersi* is ~7.5 mm (Moser & Mundy, 1996; Richards, 2005). The pelvic fin of the specimen is in contact with an unidentified larval anthozoan of ~3 mm TL.

The juvenile carangid (Figure 1f) is identified as *Caranx* cf. *latus* based on the presence of three anal‐fin spines, with a gap between the second and the third spines (Laroche et al., 1984), heavy dorsolateral and ventrolateral pigmentation, dorsal midline pigmentation extending to the nape, presence of 21 soft dorsal‐fin rays, unpigmented second dorsal‐fin and anal‐fin rays, absence of a preopercular spine and pigmentation of the first dorsal fin concentrated between the first and fourth spines (Berry, 1959). Juveniles of *C. latus* can be distinguished from *Caranx lugubris* only based on the modal number of anal‐fin rays (17 vs. 18–19, respectively), despite overlapping in total range (Laroche et al., 2005). However, the accurate count of anal‐fin rays based on the photographs was not possible, preventing proper identification to species level. Therefore, the specimen is identified as *Caranx* cf. *latus*. Available descriptions of the developmental stages of *C. latus* (Berry, 1959; Laroche et al., 2005) allow an estimate of the specimen's TL to be between 20 and 32 mm. A series of seven sequential photographs, captured by R. Collins, show the *Caranx* cf. *latus* swimming closely around a 6‐mm BL tube anemone of the family Cerianthidae.

The behaviour exhibited by some of the recorded associations was documented by the divers, and a summary of these interactions is provided below. *A. schoepfii* (Figure 1a) was photographed swimming while holding the *Palythoa* larva with its mouth. The fish was able to swim actively with the zoanthid in its mouth and demonstrated a defensive posture, moving short distances and not attempting to flee during the first two attempts of R. Collins to collect the zoanthid. In the third attempt, the *Aluterus* released the larval zoanthid and swam away. The zoanthid was undamaged, with no signs of abrasion from the holding/biting behaviour. At least four species of filefishes in adult stage were recorded mooring to benthonic invertebrates with their mouth, a behaviour referred to as ‘teeth‐anchorage’ (Eyal et al., 2011) or ‘nocturnal mooring’ (Childs, 1998) and believed to be associated with resting. Although adults may be resting while holding invertebrates with the mouth, juvenile specimens may be using zoanthids and other pelagic invertebrates for protection against predators in pelagic waters, as suggested by Johnson et al. (2025). Stromateiforms have been documented in association with cnidarians (Mansueti, 1963), ctenophores (Purcell & Arai, 2001), tunicates (Janssen & Harbison, 1981; Pastana et al., 2021, 2022) and nudibranchs (Pastana et al., 2023). Although no behavioural data were provided for the *A. regulus*‐tube anemone association (Figure 1b), the specimen was photographed swimming closely to the tentacles of the tube anemone, suggesting a well‐suited manoeuvring capacity. Similar behaviour was described for *Nomeus gronovii* (Stromateiformes, Nomeidae) and *Physalia physalis* (Jenkins, 1983). The association of *B. brama* and *I. maderensis* (Figure 1c) was terminated after an extended period of flash photography, with the fish releasing the tube anemone and swimming away. Richards (2005) described the presence of sharp teeth in larval bramids, which may be used, among other functions, to hold larval anthozoans or other pelagic invertebrates as protection against predators (e.g., Johnson et al., 2025). The photographed *B. myersi* (Figure 1e) was holding the anthozoan with their pelvic‐fin rays. Nonaka et al. (2021) described this interaction as the ‘riding’ behaviour in the context of the association of *Brama orcini* and a corymorphid hydrozoan. They observed that the pelvic‐fin rays in one *Brama* specimen were notably thickened, suggesting that it might facilitate the contact with the noxious pelagic invertebrates. However, thickened rays were not observed in other specimens of *B. orcini*. During the encounter of divers with *Caranx* cf. *latus* and the tube anemone (Cerianthidae; Figure 1f), the fish was observed swimming around the tube anemone. As the diver approached, the fish attempted to keep the anemone between itself and the camera. A similar defensive behaviour was described between *A. regulus* and nudibranchs (Pastana et al., 2023), suggesting that, in associations where fishes do not actively hold their host (biting or riding), they may use the pelagic invertebrates as a barrier against predators (Janssen et al., 1989). In contrast to the findings reported by Pastana et al. (2023), where the *A. regulus* did not leave its nudibranch even after multiple flashes and close proximity to divers, *Caranx* exhibited a different response, leaving the tube anemone and swimming away after about 1 min and seven flash photographs.

Associations between fishes and adult anthozoans can be obligatory, as in the case of anemonefishes (Da Silva & Nedosyko, 2016), or facultative and temporary, as described for the 16 other families indicated above (Feeney et al., 2019). Here we extend this relationship to epipelagic waters, describing the behaviour of early‐life stage ariommatids, bramids, carangids and monacanthids in association with larval tube anemones and zoanthids. Similar to the association in the benthos, larval and juvenile fishes seem to seek larval anthozoans in epipelagic waters for protection against predators, as some early‐stage anthozoans already possess nematocysts and other defensive structures (Stricker, 1985; Gustav Paulay personal communication). This association appears to be confined to the critical early pelagic phase, as reported for the fish‐jellyfish association (Mansueti, 1963).

Fishes in early stage of development have distinct survival strategies when interacting with pelagic invertebrates, such as hiding within them (e.g., salps; Pastana et al., 2022), mimicking potentially noxious, venomous, unpalatable and or low nutrition invertebrate models (i.e. Batesian mimicry, as described in Johnson et al., 2025) or swimming alongside and holding toxic pelagic invertebrates (Pastana et al., 2023; present study). Survival strategies characterized by a fish‐pelagic invertebrate association are frequently considered beneficial for the fish, as they seek refuge in gelatinous zooplankton in epipelagic waters, an environment naturally devoid of tridimensional complexity (e.g., Griffin et al., 2019; Johnson et al., 2025). However, benefits to the pelagic invertebrates are frequently characterized as neutral, as in the cases of commensalism (Pastana et al., 2023) and protective commensalism (Johnson et al., 2025), or even perceived to be disadvantageous, particularly in instances where the fish parasitizes or consumes the pelagic invertebrate (e.g., D'Ambra et al., 2014; Mansueti, 1963).

We suggest that the association between fish and pelagic invertebrates can be, in some cases, mutualistic, that is, potentially beneficial for the pelagic invertebrate when two criteria are met. First, both organisms must be bottom‐associated species in adulthood. Second, during early stages, the fish must associate with the larval invertebrate by holding it and swimming with it actively. Reef‐associated fishes, such as monacanthids (Kawase & Nakazono, 1994; Kingsford & Milicich, 1987), find protection against predation in tridimensional, complex, coral reefs (Griffin et al., 2019). However, these species often have an epipelagic larval stage before recruitment into reef habitats (Leis, 2010). Some anthozoans, such as zoanthids (Ryland, 1997), including *Palythoa* (Hirose et al., 2011), also have an epipelagic larval stage prior to settlement in the benthos. However, the planktonic stage of anthozoans, including the tube anemones and zoanthids identified in this study, has very limited mobility relative to fishes. The documented association of a juvenile *A. schoepfii* actively swimming while holding a zoanthid larvae, possibly *Palythoa* (Figure 1a), suggests that the fish benefits from the relationship by finding protection against predators, whereas the larval anthozoan can benefit from the active swimming of the fish as a means of dispersion. In such cases, the larval anthozoan may settle in farther from its site of origin. We offer this hypothesis based on photographic records and diver observations, but confirmation will require further experimental investigation.

The early life history of numerous marine fish species has been predominantly documented in the literature through material collected in marine surveys (e.g., Richards, 2005). However, in recent years, we have gradually seen the increase in the number of publications describing various aspects of the biology of early marine fish stages. These publications have emerged from the accumulation of information obtained through ‘blackwater’ dives [e.g., Nonaka et al., 2021; Pastana et al., 2021, 2022, 2023; Girard et al., 2024; Johnson et al., 2025; see Baldwin (2013) for information on colour patterns in larval fishes using a different methodology]. This novel source of data offers numerous insights that are previously unattainable through fixed specimens from scientific surveys, including colour in life, behaviour and association with other planktonic organisms. Therefore, we underscore the significance of community science to the advancement of ichthyology by highlighting its connection with the blackwater divers community.

## 1 SPECIMENS EXAMINED

Actinopterygii: Amarsipidae: *Amarsipus carlsbergi* (SIO 75–122; 50.4 mm SL). Ariommatidae: *Ariomma indicum* (MZUSP 123249; 129.9 mm SL); *Ariomma bondi* (MZUSP 86717; 125.3 mm SL). Balistidae: *Canthidermis maculata* (VIMS 6269; 42.9 mm SL); *Canthidermis sufflamen* (VIMS 2190; 30.9 mm SL). Bramidae: *Brama caribbea* (VIMS 33638; 134.4 mm SL); *Pterycombus brama* (VIMS 2508; 17.46 mm SL). Carangidae: *Caranx bartholomaei* (VIMS 13466; 27.8 mm SL); *Caranx crysos* (VIMS 9564; 60.7 mm FL); *Caranx hippos* (VIMS 22577; 35.5 mm FL); *C. latus* (VIMS 15080; 95.4 mm FL); *Caranx ruber* (VIMS 4627; 54.7 mm FL). Centrolophidae: *Psenopsis anomala* (MZUSP 119730; 74.2 mm SL); *Psenopsis cyanea* (MZUSP 123244; 138.6 mm SL); *Centrolophus niger* (CSIRO H 2421–01; 245.1 mm SL); *Schedophilus* sp. (MCZ 161887; 89.2 mm SL); *Seriolella porosa* (USNM 176593; 198.8 mm SL); *Tubbia tasmanica* (CSIRO H 6979–03; 325.2 mm SL); *Icichthys lockingtoni* (OS 16732; 102.3 mm SL); *Hyperoglyphe perciformis* (MZUSP 119733; 150.4 mm SL). Monacanthidae: *A. schoepfii* (VIMS 7353; 49.3 mm SL); *Aluterus scriptus* (VIMS 4725; 75.5 mm SL); *Stephanolepis hispida* (VIMS 10382; 17.1 mm SL). Nomeidae: *Psenes cyanophrys* (MZUSP 106392; 152.6 mm SL); *Psenes sio* (MZUSP 123248; 184.3 mm SL); *Cubiceps baxteri* (= reidentified to *Ariomma melana*; MZUSP 123246; 134.9 mm SL); *Cubiceps whiteleggii* (MZUSP 123247; 107.5 mm SL); *Cubiceps pauciradiatus* (MZUSP 80701; 88.6 mm SL); *Nomeus gronovii* (MZUSP 67590; 81.3 mm SL). Stromateidae: *Peprilus triacanthus* (MZUSP 123240; 128.9 mm SL); *Peprilus paru* (MZUSP 67608; 80.9 mm SL); *Pampus cinereus* (MZUSP 119734; 72.4 mm SL); *Stromateus brasiliensis* (MZUSP 51279; 136.1 mm SL). Tetragonuridae: *Tetragonurus cuvieri* (MZUSP 123241; 94.7 mm SL); Anthozoa: *Palythoa* sp. (FLMNH UFID's 14074, 14079, 14082, 14083, 16273, 17328); *I. maderensis* (FLMNH UFID's 13821, 13852, 14076, 14217, 14222, 14385); *Isarachnanthus* sp. (FLMNH UFID's 13443, 13429, 13439, 13452, 13859); Cerianthidae (FLMNH UFID's 14212, 14218, 15271, 16505).

## AUTHOR CONTRIBUTIONS

Gabriel V. F. Afonso: data collection, figure preparation, manuscript writing, manuscript editing and literature review. G. David Johnson: data collection and literature review. R. Collins: data collection, manuscript editing and literature review. Murilo N. L. Pastana: data collection, manuscript writing, manuscript editing and literature review.

## FUNDING INFORMATION

Gabriel V. F. Afonso: Leonard P. Schultz Short‐term Fellowship, and VIMS Office of Academic Affairs and Research Section for Natural Resources. G. David Johnson: Herbert R. and Evelyn Axelrod Endowment Fund for Systematic Ichthyology. Murilo N. L. Pastana: Sara E. and Bruce B. Collette Postdoctoral Fellowship in Systematic Ichthyology, and Programa de Apoio a Novos Docentes of Pró‐Reitoria de Pesquisa e Inovação (PRPI), University of São Paulo.
